# Verruciform Xanthoma Within the Cyst Lining of Hidradenitis Suppurativa

**DOI:** 10.1111/cup.70042

**Published:** 2025-12-16

**Authors:** Deaquan Nichols, Aref Moshayedi, Ja Hea Gu, David M. Milgraum, Mark C. Mochel

**Affiliations:** ^1^ School of Medicine Virginia Commonwealth University Richmond Virginia USA; ^2^ Department of Dermatology Virginia Commonwealth University Health System Richmond Virginia USA; ^3^ Division of Plastic and Reconstructive Surgery, Department of Surgery Virginia Commonwealth University Health System Richmond Virginia USA; ^4^ Department of Pathology Virginia Commonwealth University Health System Richmond Virginia USA

**Keywords:** cyst, hidradenitis suppurativa, verruciform xanthoma

## Abstract

Verruciform xanthoma (VX) is a rare lesion most often seen in the oral mucosa or anogenital region, most commonly characterized histologically by verrucous epithelial hyperplasia and foamy histiocytes in the papillary dermis. While VX has been reported in association with inflammatory dermatoses such as lichen planus and lichen sclerosus, its occurrence in the context of hidradenitis suppurativa (HS) has not been previously documented. In this case we present a 56‐year‐old man with long‐standing, Hurley Stage 3 HS affecting the gluteal region. Following surgical excision of a draining sinus tract, histopathology revealed a squamous‐lined follicular cyst consistent with HS, with approximately 10% of the cyst lining displaying features characteristic of VX, including papillomatous acanthosis, parakeratosis with neutrophils, and underlying foamy macrophages. Retrospective review of prior HS specimens did not show similar changes. This case expands the histologic spectrum of HS and suggests that chronic inflammation may promote secondary verruciform xanthomatous changes within follicular cysts. Although mutations in the cholesterol biosynthesis gene NSDHL have been linked to VX, no lichenoid or syndromic features were observed in our patient, supporting an inflammatory rather than genetic etiology. Recognition of VX‐like changes in HS is important to avoid misdiagnosis as squamous cell carcinoma and further elucidates the complex epithelial remodeling in chronic inflammatory dermatoses.

## 1 Introduction

Verruciform xanthoma (VX) most commonly presents as a lesion of oral mucosa or anogenital skin and displays distinctive histopathology, including verrucous hyperplasia with underlying foamy histiocytes [1]. VX has been reported in association with a small number of inflammatory conditions, including lichen planus and lichen sclerosus [2]. Herein, we report a case of hidradenitis suppurativa (HS) with histopathologic changes of VX within a follicular cyst.

## 2 Case Report

**FIGURE 1:**
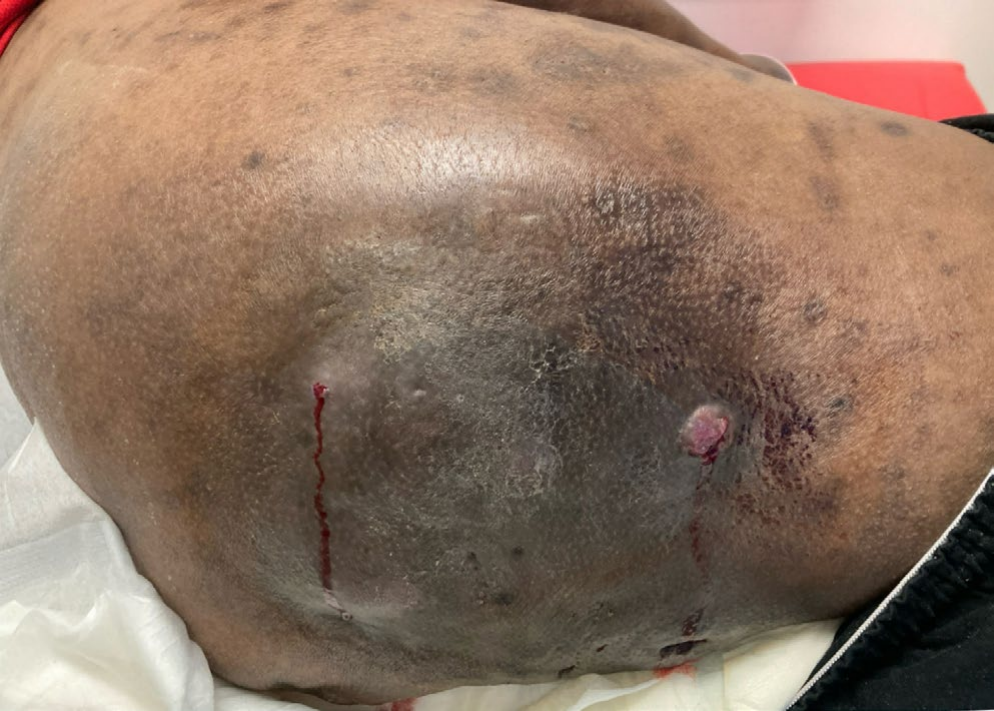
Pre‐operation lesion on lateral, posterior right gluteal region prior to operation.

A 56‐year‐old male with a history of peripheral arterial disease, type 2 diabetes mellitus, hyperlipidemia, end‐stage renal disease, and longstanding HS of Hurley Stage 3 presented for treatment of HS. The patient's chronic, painful HS lesions in the gluteal and genital regions had been previously managed with chlorhexidine wash, doxycycline, and clobetasol. Clinical examination revealed an 8 × 5 cm indurated lesion with a tunneling, draining sinus tract involving the lateral, posterior right gluteal region (Figure 1). Excision and debridement were performed, and the specimen was sent for pathological analysis.

**FIGURE 2:**
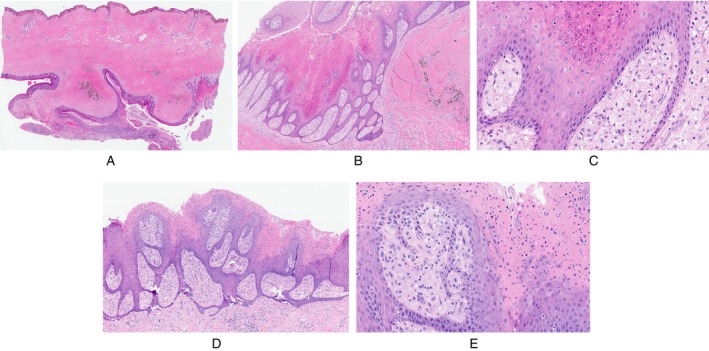
Histopathology of the excisional specimen with dermal and subcutaneous fibrosis with a deep, complex, squamous‐lined, and keratin‐containing cystic lesion (A, 10×, H&E). The lining focally showed papillomatous hyperplasia with wedge‐shaped parakeratosis (B, 100×, H&E) with papillary dermal foamy macrophages (C, 400×, H&E), indicative of verruciform xanthoma. These features were multifocal, and the parakeratosis was associated with neutrophils in some foci (D and E, 100× and 400×, H&E).

Histopathological examination of the excisional specimen revealed dense dermal and subcutaneous fibrosis with a complex squamous‐lined cyst containing orthokeratin and associated with foci of disruption and granulation tissue (Figure 2A). Most of the cyst showed acanthotic squamous epithelium, typical for follicular cysts seen in HS. However, approximately 10% of the squamous cyst lining showed acanthosis with papillomatosis, intervening wedge‐shaped parakeratosis containing neutrophils, and underlying papillary dermal aggregates of foamy macrophages, consistent with incidental changes of VX (Figure 2B,C). Discontinuous foci with similar changes of VX were noted (Figure 2D,E). The follicular, inflammatory, and fibrotic changes were consistent with hidradenitis suppurativa, while the changes of VX were favored to represent a secondary process involving the pre‐existing cyst. Significant lichenoid changes were not seen in the specimen. Clinically, the patient did not have significant cutaneous lesions, for example, epidermal nevus, beyond those of HS. Retrospective histopathologic review of excisional specimens of HS lesions affecting the left groin from 6 years prior did not show similar VX changes in the cyst lining.

## 3 Discussion

The histopathology of HS includes a spectrum of changes, including cystically dilated follicular canals, neutrophilic abscesses, granulation tissue, and fibrotic scar tissue with lymphohistiocytic and lymphoplasmacytic inflammation [3]. Cystic hair follicles are lined by follicular infundibulum‐like squamous epithelium and may show continuity with the epidermis and other cystically dilated follicles. Similarly, sinus tracts connect to the surface and other tracts but are lined by granulation tissue [3]. Changes to the epithelial lining of the cystic follicles in HS include variable degrees of hyperplasia and occasional disruption at the site of cyst rupture. More broadly, spontaneous follicular infundibular cysts (also termed “epidermal inclusion cysts”) have been reported to show a variety of changes, including verrucous hyperplasia [4] and epidermolytic hyperkeratosis [5]. To the authors' knowledge, features of VX have not been reported in the context of HS pathology or follicular infundibular cyst. VX characteristically shows verrucous epithelial hyperplasia with highly eosinophilic parakeratosis with wedge‐shaped indentations into the spinous layer, hypogranulosis, intraepithelial neutrophils, and papillary dermal aggregates of foamy macrophages [6]. The squamous epithelial changes, memorably likened to a “wart on fire” by Rush and Bennett, often permit rapid recognition of the lesion at scanning magnification [7]. The presence of an endophytic squamous proliferation with confluent parakeratosis and granular zone loss could raise consideration of squamous cell carcinoma, an uncommon complication of chronic HS lesions, although the distinctive wedge‐shaped parakeratosis, papillary dermal macrophages, and lack of significant cytologic atypia allow for straightforward diagnosis of VX. A few reports have documented VX with a cystic arrangement, although these have been endophytic cystic proliferations lined entirely by VX, contrasting the case presented here with focal changes of VX within an otherwise typical follicular infundibular cyst [8].

While the pathogenesis of VX is not completely understood, genetic and inflammatory factors likely contribute. Current hypotheses for pathogenesis include localized disruption of lipid metabolism and pathologic macrophage‐predominant response to injury [1]. VX may present in the Congenital Hemidysplasia with Ichthyosiform Erythroderma and Limb Defects (CHILD) syndrome, an X‐linked dominant multisystem disease secondary to mutations in the cholesterol synthesis gene *NSDHL* [9]. Rare studies have identified mutations in the same gene in approximately half of sporadic oral VX and in a minority of sporadic cutaneous VX [9, 10]. Among other uncommon associations, VX may also arise within lichen planus and lichen sclerosus, suggesting an inflammatory pathway to VX development [2]. One report detailed VX arising on penile and perineal skin years after skin grafting for necrotizing fasciitis [11]. While a mutation‐driven pathogenesis in our case is possible, we favor that the inflammatory milieu of HS resulted in the discontinuous foci of VX.

In summary, histopathologic changes of VX may appear in the cystically dilated follicular epithelium of HS. In addition to adding to the literature regarding inflammatory conditions that may give rise to VX, our report highlights an unusual histopathologic feature of HS that possibly could mimic a squamous cell carcinoma.

## Funding

The authors have nothing to report.

## Ethics Statement

This study was conducted in accordance with the ethical standards of the institutional review board; all patient data were de‐identified to ensure confidentiality.

## Conflicts of Interest

The authors declare no conflicts of interest.

## Data Availability

The data that support the findings of this study are available from the corresponding author upon reasonable request.
